# Sulfonated and Carboxymethylated β-Glucan Derivatives with Inhibitory Activity against Herpes and Dengue Viruses

**DOI:** 10.3390/ijms222011013

**Published:** 2021-10-12

**Authors:** José Louzinho Lopes, Vinicius Seiki Takemura Quinteiro, Jéssica Wouk, Maria Laura Darido, Robert F. H. Dekker, Aneli M. Barbosa-Dekker, Václav Vetvicka, Mário A. A. Cunha, Ligia Carla Faccin-Galhardi, Alexandre Orsato

**Affiliations:** 1 Laboratory of Synthesis of Medicinal Molecules (LaSMMed), Department of Chemistry, CCE, Universidade Estadual de Londrina, Londrina CEP 86057-970, Parana, Brazil; louzinho.lopes254@gmail.com (J.L.L.); vinicius.seiki@uel.br (V.S.T.Q.); anelibarbosa@gmail.com (A.M.B.-D.); 2 Faculdade de Ciências da Saúde, Universidade Zambeze, Tete CP 2301, Mozambique; 3 Laboratory of Virology, Department of Microbiology, CCB, Universidade Estadual de Londrina, Londrina CEP 86057-970, Parana, Brazil; je_wouk@hotmail.com (J.W.); marialaura.98@outlook.com (M.L.D.); lgalhardi@uel.br (L.C.F.-G.); 4 Beta-Glucan Produtos Farmoquímicos EIRELI, Lote 24A, Bloco Zircônia, Universidade Tecnológica Federal do Paraná, Câmpus Londrina, Londrina CEP 86036-700, Parana, Brazil; xylanase@gmail.com; 5 Department of Pathology and Laboratory Medicine, University of Louisville, Louisville, KY 40292, USA; vaclav.vetvicka@louisville.edu; 6 Department of Chemistry, Universidade Tecnológica Federal do Paraná, Pato Branco CEP 85503-390, Parana, Brazil; mario.utfpr@gmail.com

**Keywords:** (1→3)(1→6)-β-D-glucan, antivirals, anionic polysaccharides, herpes simplex virus, dengue virus

## Abstract

The infection of mammalian cells by enveloped viruses is triggered by the interaction of viral envelope glycoproteins with the glycosaminoglycan, heparan sulfate. By mimicking this carbohydrate, some anionic polysaccharides can block this interaction and inhibit viral entry and infection. As heparan sulfate carries both carboxyl and sulfate groups, this work focused on the derivatization of a (1→3)(1→6)-β-D-glucan, botryosphaeran, with these negatively-charged groups in an attempt to improve its antiviral activity. Carboxyl and sulfonate groups were introduced by carboxymethylation and sulfonylation reactions, respectively. Three derivatives with the same degree of carboxymethylation (0.9) and different degrees of sulfonation (0.1; 0.2; 0.4) were obtained. All derivatives were chemically characterized and evaluated for their antiviral activity against herpes (HSV-1, strains KOS and AR) and dengue (DENV-2) viruses. Carboxymethylated botryosphaeran did not inhibit the viruses, while all sulfonated-carboxymethylated derivatives were able to inhibit HSV-1. DENV-2 was inhibited only by one of these derivatives with an intermediate degree of sulfonation (0.2), demonstrating that the dengue virus is more resistant to anionic β-D-glucans than the Herpes simplex virus. By comparison with a previous study on the antiviral activity of sulfonated botryosphaerans, we conclude that the presence of carboxymethyl groups might have a detrimental effect on antiviral activity.

## 1. Introduction

Anionic polysaccharides have attracted much attention over the past decades for their inhibitory activity against viruses [1,2]. Among these, the sulfated polysaccharides, mainly from seaweeds, stand out as showing potent activity against several enveloped viruses, such as Herpes simplex (HSV-1 and HSV-2) [3,4], influenza [5], HIV-1 and 2 [1,5,6], and Dengue (DENV 1-4) [7,8]. The latest studies revealed that heparin, a sulfated polysaccharide, inhibits cellular invasion by SARS-CoV-2, causative of Coronavirus disease (COVID-19) [9]. The elucidation of the infection mechanism of enveloped viruses paved the way for the discovery of these inhibitors of viral infection, natural molecules belonging to the class of carbohydrates [5,10].

Herpes symptoms range from ulcerative and vesicular lesions to encephalitis; a severe complication [11]. Drugs currently used to treat HSV-1 infection are acyclovir and its analogs, foscarnet, and cidofovir. However, the number of strains resistant to these drugs is increasing [12]. On the other hand, there is no specific treatment approved for dengue. The therapy of dengue patients, regardless of the severity of the disease, remains symptomatic. About three-quarters of infected people are asymptomatic, while others can experience fever and pain, and more severe symptoms like a hemorrhagic fever that is considered rare. The long-lasting development of anti-DENV vaccines still experiences problems regarding all serotype efficacies [8]. Both diseases affect millions of people all over the world according to the World Health Organization [13,14].

Sulfated polysaccharides offer a promising alternative as antiviral drugs for their therapeutics. They inhibit the first step of infection, where the glycoprotein on the viral envelope utilizes its positive charges to interact with negative charges of heparan sulfate (HS), one of the host cell surface receptors. By inhibiting this step, sulfated polysaccharides mimic HS, thus blocking the virus from entering into the host cell [5,15].

Examples of naturally sulfated polysaccharides, all extracted from red seaweeds, with potent antiviral activity are agarans [15], galactans [3,16], and carrageenans [4,17]. Despite their biological potential against enveloped viruses, sulfated polysaccharides in seaweeds are only seasonally available, which limits their production of different amounts of polysaccharides throughout the year [18]. Moreover, their purification from the carbohydrate extracts is a laborious process [17]. Thus, the present work is dedicated to the chemical derivatization of a fungal polysaccharide, which overcomes these disadvantages.

Botryosphaeran (BOT) is an exopolysaccharide (EPS) of the (1→3)(1→6)-β-D-glucan type produced by the fungus *Botryosphaeria rhodina* MAMB-05 through a submerged fermentation process [19]. Previously, our research group reported that sulfonated botryosphaerans exhibited potent antiviral activity against HSV and DENV [20]. Therefore, to make botryosphaeran more similar to HS, the host cell receptor-bearing carboxyl and sulfonate groups, we proposed in the present study using a botryosphaeran preparation containing both carboxymethylated and sulfonated derivatives. This hypothesis was assessed on the antiviral evaluation of these derivatives against HSV-1 (strains KOS and AR, sensitive and resistant to acyclovir, respectively) and DENV-2, enveloped viruses, and poliovirus, a non-enveloped virus.

## 2. Results and Discussion

### 2.1. Preparation of Carboxymethylated and Sulfated Derivatives

**Figure 1:**
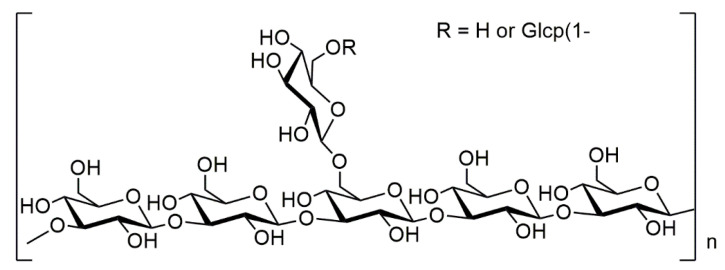
Representative chemical structure of botryosphaeran.

Botryosphaeran consists of a backbone chain comprising (1→3)-linked β-D-glucose units branched at carbon-6 with glucose or gentiobiose through β-(1→6)-linkages (Figure 1). The extent of branching is 21%, a substituent on every 5 glucose units along the backbone chain [19].

**Scheme 1:**
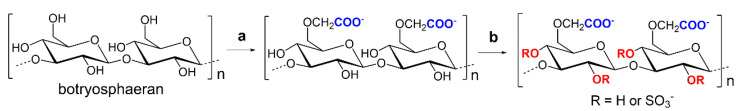
Preparation of sulfonated/carboxymethylated derivatives of BOT. Reaction step (**a**) ClCH_2_COOH, isopropanol, aqueous NaOH (20%), room temperature to 60 °C, 4 h, and (**b**) ClSO_3_H, pyridine, formamide, −4 °C or 60 °C, 3–26 h. For simplification purposes, the polysaccharide structure was represented without branching.

Botryosphaeran mimetics of HS were prepared by carboxymethylation followed by sulfonylation (Scheme 1). The reason for this order was to bind the carboxymethyl groups to the O atom of the more reactive OH-6 group of glucosyl units of BOT, to mimic the carboxyl groups found in the C-6 position of uronic acid units of HS. The carboxymethyl groups were introduced by reacting BOT with monochloroacetic acid (MCA) in an alkali solution [21,22]. The presence of carboxyl groups was quantified by an acid-base titration method that resulted in a degree of substitution by carboxymethylation (DS_CM_) of 0.9, calculated from the formula in Section 3.2. This value indicates that for every ten glucose units of the polysaccharide, an average of nine units were functionalized with carboxymethyl groups. All sulfonated derivatives were prepared from this carboxymethylated BOT (CM-BOT).

Sulfonylation of the carboxymethylated derivative was carried out with chlorosulfonic acid, pyridine, and formamide as solvent. Three sulfonated samples were prepared after two cycles of sulfonylation by varying the time, temperature, and the proportion of chlorosulfonic acid (CM-S-BOT-1, CM-S-BOT-2, and CM-S-BOT-3; the specific reaction conditions are mentioned in Section 3.1.3). The degree of substitution of the sulfonated derivatives (DS_S_) CM-S-BOT-1, CM-S-BOT-2, and CM-S-BOT-3 were 0.1, 0.2, and 0.4, respectively (calculated from the formula in Section 3.2). These DS_S_ values were much lower than that of DS_CM_. These results are in agreement with literature values that report that the substitution of the primary alcohols by carboxymethyl groups can hamper the sulfonylation step due to the steric hindrance effect caused by this group on the secondary alcohol positions [23]. This is most likely the main reason why the DS_S_ of the sulfonated/carboxymethylated derivatives are much lower when compared to sulfonated botryosphaeran from a previous report, with DS_S_ ranging from 0.4 to 1.1 [20].

**Figure 2:**
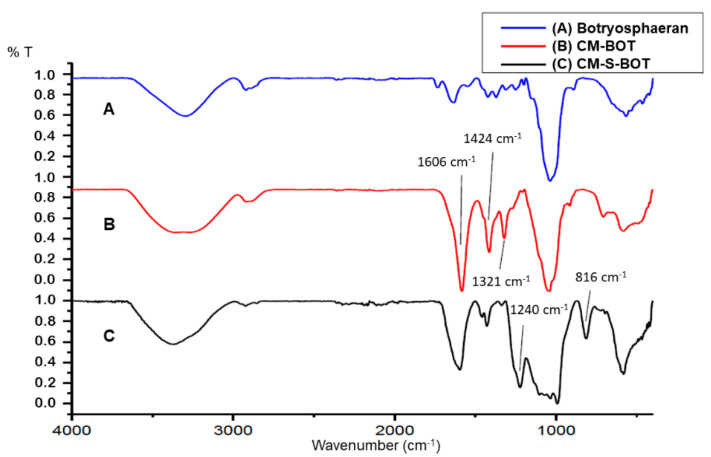
Comparison between FT-IR spectra of BOT (**A**) and its carboxymethylated (**B**, CM-BOT) and sulfated/carboxymethylated (**C**, CM-S-BOT) derivatives.

The FT-IR spectrum of native BOT (Figure 2A) was compared to the spectrum of the carboxymethylated derivative CM-BOT (Figure 2B). Both spectra exhibited bands typical of polysaccharides in the region between 4000 and 500 cm^−1^. The broadband between 3500 and 3000 cm^−1^ was attributed to the stretching vibration of the OH groups. The strong band at 1037 cm^−1^ corresponds to the C–O stretching of alcohols and glucose rings. The band at 1640 cm^−1^ of the BOT spectrum corresponds to the stretching vibration of the glucose ring. After carboxymethylation, two strong absorption bands appeared in the spectrum (Figure 2B) at 1606 and 1424 cm^−1^, and were assigned to COO^–^ asymmetric and symmetric stretching vibrations, respectively. These results indicated that BOT was successfully carboxymethylated.

The sulfonated derivatives were also characterized by FT-IR spectroscopy (Figure 2C) and their respective spectra were compared to that of CM-BOT and underivatized BOT. The appearance of two new bands at 1240 and 816 cm^−1^, attributed to S=O asymmetrical and C–S–O symmetrical vibrations, indicates the presence of sulfonate groups bound to the polysaccharide and, therefore indicates the success of the sulfonylation reaction.

**Figure 3:**
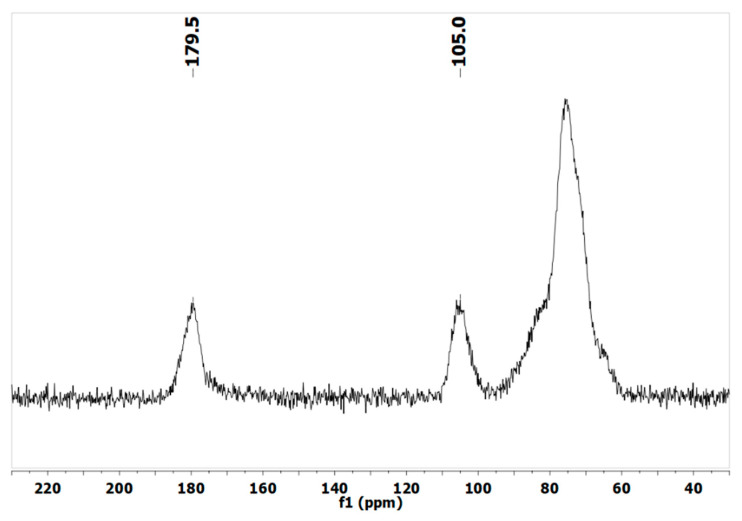
Solid-state ^13^C NMR spectrum of carboxymethylated botryosphaeran (CM-BOT).

**Figure 4:**
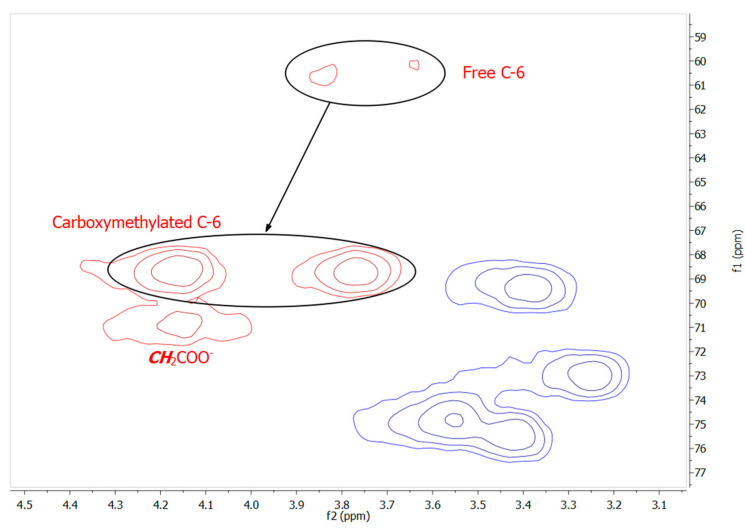
^1^H-^13^C HSQC spectrum of CM-BOT (C-6 region). Free C-6, carboxymethylated C-6, and carboxymethyl methylene group (*CH_2_*COO^–^) signals are indicated to show the substitution pattern of CM-BOT. (full spectrum is shown in the Supplementary Material Figure S1).

Preliminary structural analysis of the carboxymethylated derivative (CM-BOT) was performed by solid-phase ^13^C NMR (Figure 3) and showed a peak between 170–190 ppm. The strong signal at 179.5 ppm was attributable to carbonyl groups and indicates the presence of carboxymethyl groups along the polysaccharide chain, and confirms the data from FT-IR analysis. The signal at 105 ppm belongs to C-1 of glucose units and the broad signal between 90–60 ppm was attributed to the glucosyl non-anomeric carbon atoms. Two-dimensional NMR analysis (HSQC, Figure 4) provided strong evidence for predominant OH-6 carboxymethylation as the original C-6 signal of BOT (60.6 ppm) almost disappeared and a new signal for C-6 emerged downfield (68.7 ppm). A new signal at 70.9 ppm was assigned to the carboxymethyl methylene group, to confirm the derivatization. Signals of other C-atoms shifted only slightly from previously reported assignments [19,20,24].

**Figure 5:**
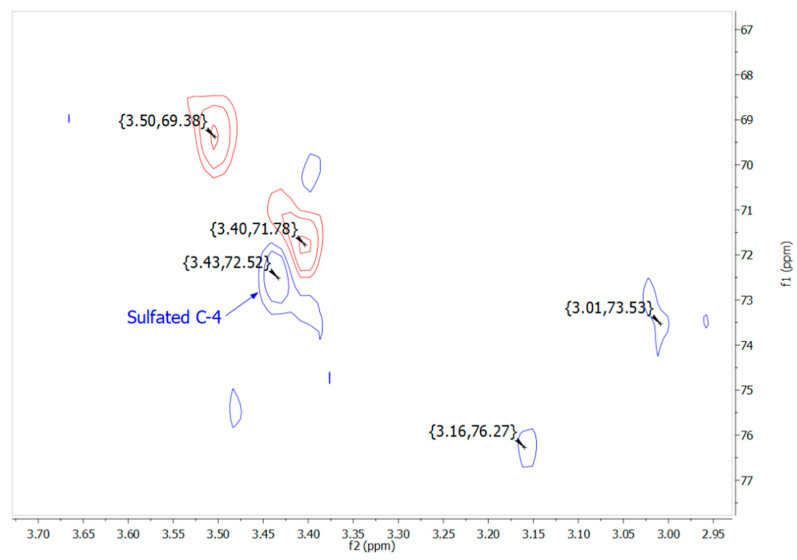
^1^H-^13^C HSQC spectrum of CM-S-BOT-1. Region of non-anomeric glucosyl carbon atoms is shown. (full spectrum is shown in the Supplementary Material Figure S2).

NMR analysis of CM-S-BOT-1 revealed no signal around 68.7 ppm, where the C-4 NMR signal of the →3)-Glc*p*-(1→ glucose units of native BOT usually appear [19]. The appearance of a ^13^C signal at 72.5 ppm, attributed to C-4, indicated that OH-4 was probably the main site of sulfonylation (Figure 5). Substitution at this position might predominate over OH-2 substitution due to less steric hindrance caused by the presence of glucosyl residues at C-1 and C-3 positions. In addition, a new signal at 62.8 ppm indicated that OH-6 sulfonylation also occurred (see HSQC, Supplementary Material Figure S2). Therefore, the substitution pattern of CM-S-BOT samples consists of carboxymethylation of OH-6 and predominant sulfonylation of OH-4 and OH-6.

### 2.2. Cytotoxicity and Antiviral Activity

All of the BOT derivatives did not display cytotoxicity in Vero cells, up to the highest concentration tested (100 μg mL^−1^). In a previous study, native BOT exhibited low cytotoxicity (CC_50_ > 100 μg mL^−1^) and showed moderate activity against HSV-1: 100%, 52%, 38%, and 29% of viral inhibition at 100, 50, 25, and 12.5 μg mL^−1^, respectively, for HSV-KOS (IC_50_ 39.3 μg mL^−1^), and 69.6%, 55%, 31.4%, and 0%, at same concentrations for HSV-AR (IC_50_ 47.5 μg mL^−1^) [20].

The results of the antiviral activity of BOT and its derivatives against HSV-1 are summarized in Table 1. The carboxymethylated BOT derivative (CM-BOT) did not exhibit anti-HSV-1 and anti-DENV-2 activities and agreed with literature reports. Serotype DENV-2 has a higher prevalence in Brazil, and according to the Brazilian Health Ministry is mainly responsible for most diagnosed cases of dengue [25]. Möller et al. (2012) reported that the presence of carboxymethyl groups on hyaluronan derivatives did not contribute to antiviral activity against HSV-1 [26]. The introduction of carboxymethyl groups on BOT apparently changed its triple-helix conformation [27], and, consequently, the hydrophobic interactions responsible for antiviral activity have been implicated [28,29]. Moreover, the removal of sulfate groups from heparan sulfate made it inactive for HSV binding, despite the presence of carboxyl groups over its chain [10]. Similarly, the negative charges introduced by carboxymethylation of BOT might not be sufficient to confer HSV binding to CM-BOT.

Alternatively, all sulfonated derivatives of CM-BOT (i.e., CM-S-BOT) inhibited HSV-KOS and HSV-AR at low-to-high concentrations (IC_50_։ 12.4 to >100 μg mL^−1^). Against HSV-AR, the IC_50_ values of these derivatives decreased with an increase in their DS_S_, suggesting that more sulfonated derivatives of BOT were more potent against HSV-AR, as previously reported [20]. CM-S-BOT-2 and -3 were more potent than BOT alone against this viral strain. Surprisingly, the IC_50_ values of these derivatives for HSV-KOS increased with higher DS_S_ values. While CM-S-BOT-1 and -2 were more potent than BOT, CM-S-BOT-3 showed no activity against HSV-KOS. Similar results were found for HSV-KOS by Jana et al. (2021) [30] for arabinogalactan. The reason for this lack of activity remains unknown. The IC_50_ values of our sulfonated derivatives of CM-BOT were higher than the BOT sulfonated derivatives reported in our previous work (IC_50_ ranged from 2.4 to 7.3 μg mL^−1^), indicating that the present compounds were less potent. For example, a sulfonated BOT derivative (DS_S_ 0.4, IC_50_ 7.3 μg mL^−1^, SI 68.5) reported by Sacchelli et al. (2019) [20] was found to be more active against HSV-AR compared to its equally sulfonated counterpart, CM-S-BOT-3 (DSs 0.4, IC_50_ 25.7 μg mL^−1^). These results lead us to assume that the bulkiness of the carboxymethyl groups might harm the interaction between sulfonate groups of polysaccharides and the viral envelope glycoproteins.

Regarding the anti-dengue evaluation, only derivative CM-S-BOT-2, with DS_S_ 0.2, exhibited antiviral activity against serotype DENV-2 (IC_50_ 64.0 µg mL^−1^), demonstrating that when carboxyl and sulfonate groups were both present in the polysaccharide structure, the degree of substitution by sulfonate groups directly influenced antiviral activity. The absence of inhibition in CM-S-BOT-1 and CM-S-BOT-3 suggests that a lesser or greater degree of substitution by sulfonate groups, respectively, may have a detrimental effect on anti-DENV activity. By comparing anti-HSV with anti-DENV results, including those from our previous work [20], it is possible to affirm that the dengue virus was more resistant to inhibition by anionic β-D-glucans than the Herpes simplex virus.

Despite the lower antiviral activity of our sulfonated-carboxymethylated derivatives in comparison with sulfonated polysaccharides reported in the literature [31,32,33,34], our results demonstrated that the presence of sulfonate groups on a carboxymethylated β-glucan mostly enhanced its antiherpetic activity. In another study, sulfonated-carboxymethylated hyaluronan derivatives with DS_S_ < 1.0 were not able to inhibit HSV-1 [26]. Our BOT derivatives inhibited this virus even at a minimum degree of substitution of DS_S_ 0.1. In addition, they were mostly better HSV inhibitors than the parent polysaccharide, BOT (Table 1). The acidic sulfonate and carboxymethyl groups present in the derivatized β-glucan structure enhanced its antiherpetic activity, probably by increasing its affinity for the viral glycoproteins.

**Figure 6:**
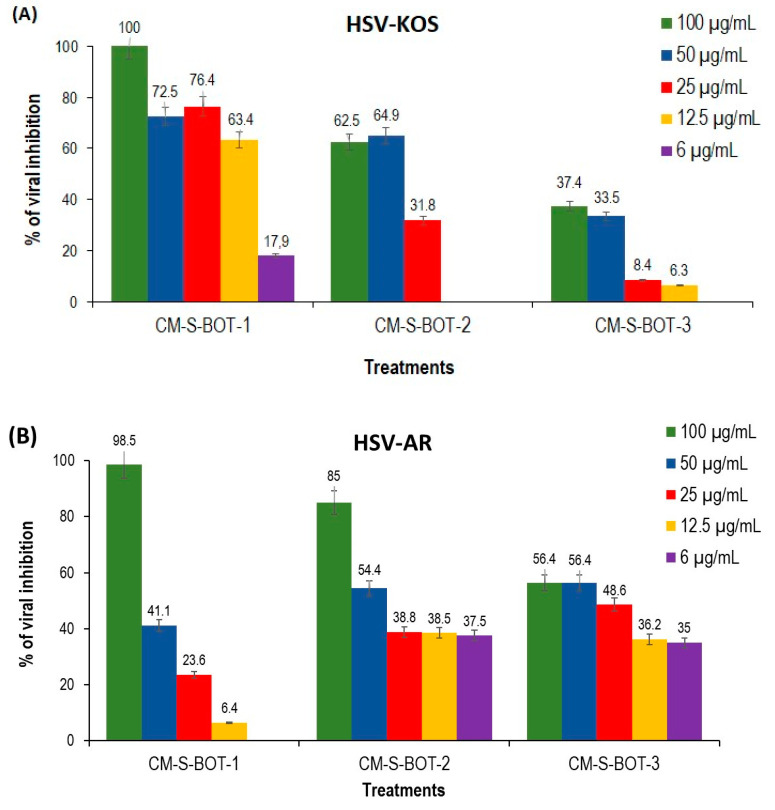
Inhibition of HSV-KOS (**A**) and HSV-AR (**B**) by decreasing concentrations of CM-S-BOT-1, CM-S-BOT-2, and CM-S-BOT-3. Mean ± standard error of the mean (*n* = 6); The differences in % of viral inhibition were statistically significant compared to infected and untreated cells (*p* < 0.05).

As observed in Figure 6, all sulfonated-carboxymethylated derivatives exhibited antiherpetic activity in a dose-dependent manner. At 100 μg mL^−1^, both HSV-KOS and HSV-AR were completely inhibited by CM-S-BOT-1 and was the only derivative that inhibited HSV-KOS (17.9% inhibition) at the lowest concentration tested (6.25 μg mL^−1^). However, HSV-AR inhibition by CM-S-BOT-1 fell dramatically with decreasing concentrations, to exert no inhibition at 6.25 μg mL^−1^, while CM-S-BOT-2 and -3 inhibited the virus to the extent of 37.5% and 35.0% at this concentration, respectively. The inhibition of HSV-AR by CM-S-BOT-2 and -3 did not fall substantially at decreasing concentrations of these derivatives, demonstrating that they preserved antiviral activity even at lower concentration levels. HSV strains can acquire resistance to Acyclovir, such as HSV-1 AR, due to mutations in the genes of thymidine kinase (95%) or viral DNA polymerase (5%) [35]. The sequencing of the *UL23* and *UL30* genes, respectively, allows the determination of HSV resistance. However, other undescribed mutations may also be present, influencing the phenotypic differences between strains [36]. This could explain the difference in inhibition found between the strains for each sulfonated CM-BOT derivative, with less discrepancy for CM-S-BOT-2.

As expected, all of the derivatives were inactive against Poliovirus, a non-enveloped virus. Taken together, the present results suggest that, like other sulfonated polysaccharides, our sulfonated-carboxymethylated botryosphaerans may inhibit HSV-1 and, to a lesser extent, DENV-2 by interacting with the viral envelope glycoproteins. The present BOT derivatives bear negative charges, provided by the carboxyl and sulfonate groups that would interact with positive charges located in basic amino acid-rich motifs of the viral glycoproteins. These are the same motifs that interact with the host cell viral receptor, heparan sulfate, before viral entry into the cell. As heparan sulfate also bears carboxyl and sulfate groups, our derivatives would mimic this cell receptor, and inhibit viral entry by blocking the interaction between the enveloped virus and heparan sulfate. A limitation of our design is the fact that carboxyl groups of heparan sulfate are provided by uronic acid residues while in the present BOT derivatives, they are provided by carboxymethyl groups. This difference results in more distancing of the carboxyl groups from the polysaccharide chain, which might reduce the interaction of sugar rings with viral envelope glycoproteins and slightly compromise the antiviral potential of these BOT derivatives. To overcome this limitation, sulfonated BOT derivatives bearing glucuronic acid residues are currently under development and antiviral evaluation in our research group.

## 3. Materials and Methods

Monochloroacetic acid, chlorosulfonic acid, formamide, pyridine, trichloroacetic acid, MTT (3-(4,5-dimethylthiazol-2-yl)-2,5-diphenyltetrazolium bromide), and MTT solubilization solution (10% Triton X-100 in acidic isopropanol) were purchased from Sigma-Aldrich (St. Louis, MO, USA). Acyclovir (ACV, 10 μg mL^−1^) was obtained from Novafarma Ind. Farmac. (Anápolis-GO, Brazil). All chemicals were of analytical grade.

### 3.1. Production of the Compounds

#### 3.1.1. Botryosphaeran Production

BOT was produced by the ascomyceteous fungus *Botryosphaeria rhodina* isolate MAMB-05 cultivated on a medium containing sucrose under submerged fermentation conditions, and isolated from the cell-free fermentation broth by precipitation with ethanol [19,37]. The precipitate (BOT) recovered was re-solubilized in water, followed by dialyzing against distilled water for 48 h and lyophilized. The dried powder was stored at −20 °C until required.

#### 3.1.2. Preparation of Carboxymethylated BOT (CM-BOT)

In a round-bottom flask containing 0.106 g of native BOT, 4 mL of isopropanol were added and the mixture was stirred at room temperature (RT) for 15 min. Then, 1.4 mL of aqueous NaOH (20%, *w*/*v*) was added dropwise. The resulting mixture was vigorously stirred at RT for 1 h, followed by the addition of 0.5 mL of monochloroacetic acid (4 mol L^−1^), and the reaction mixture was heated in an oil-bath (60 °C) whilst stirring for 4 h. Thereafter, the reaction mixture was neutralized with aqueous HCl (0.1 mol L^−1^). The mixture was then dialyzed exhaustively against distilled water, evaporated, and dried by lyophilization.

#### 3.1.3. Preparation of Sulfonated Derivatives of CM-BOT (CM-S-BOT)

In a round-bottom flask containing CM-BOT, formamide and pyridine (1:1, both dried over molecular sieves) were added under an inert atmosphere (Argon), in the proportion of 1 mL of the above solvent mixture per 2.5 mg of CM-BOT, and the resulting mixture stirred at RT for 24 h. Then, 0.8–4.0 mL of chlorosulfonic acid (100–296 equivalents/glucose unit) was added dropwise to the flask in an ice bath during the time of 1 h. After the addition, the flask was stirred at approximately −4 °C for 3–26 h.

In another experimental condition, the flask was kept under stirring at 60 °C in an oil-bath for 4 h. After this time, the reaction was stopped with the addition of approximately 50 mL of iced-distilled water and neutralized with a saturated aqueous NaHCO_3_ solution. The specific conditions for obtaining three sulfonated samples were as follows: CM-S-BOT-1 (226 equiv. of ClSO_3_H, −4 °C and 3 h); CM-S-BOT-2 (100 equiv. of ClSO_3_H, 60 °C, and 4 h); CM-S-BOT-3 (296 equiv. of ClSO_3_H, −4 °C, and 26 h).

### 3.2. Quantification of Carboxymethyl Groups (DS_CM_) and Sulfonate Groups (DS_S_)

The degree of substitution (DS_CM_) of the carboxymethylated derivatives was determined by acid-base titration, and calculated according to the following equations [38,39]:

Wc=(c×Mc×(Vb−Vs)×100%)/m

DScm=Wc×Ma/(100%−Wc)×Mc

where Wc is the carboxymethyl group content of the CM-BOT sample (%, *w*/*w*); c is the concentration of HCl used for titration (mol L^−1^); Mc is the molar mass of the carboxymethyl functional group reacting with BOT (58 g mol^−1^); Ma is the molar mass of an anhydroglucose unit (162 g/mol); Vb is the volume of aqueous HCl used for blank titration (mL); Vs is the volume of aqueous HCl used for sample titration (mL); m is the weight of the BOT sample (mg), and DS_CM_ is the degree of carboxymethyl substitution in the CM-BOT sample.

The degree of substitution by sulfonylation (DS_S_), representing the sulfonate content, was determined by the turbidimetric method of Dodgson and Price [40], and calculated according to the following equations:

$$S\%={\mathrm{SO}}_{4}^{2-}\left(\mu g\right)\frac{0.1374\times 100}{m}$$

$$\mathrm{DSs}=\frac{162.14\times S\%}{3200-102\times S\%}$$

where S% is the sulfur content, m is the weight of the BOT sample (µg) and DS_S_ is the average number of sulfonate groups per glucose unit of the polysaccharide.

Fourier Transform Infra-red (FT-IR) spectra were recorded on a Bruker Vertex 70 spectrometer (Bruker, Berlin, Germany) with attenuated total reflectance accessory (Platinum ATR), within the range 4000–400 cm^−1^.

For solid-state NMR analysis, the polysaccharide samples were added in a 4 mm rotor and subjected to analysis using a CP-MAS NMR probe with a rotation of 10k Hz, using Bruker standard pulse. Solid-state and 2D NMR spectra were acquired on a Bruker Avance III 400 MHz NMR spectrometer (Bruker, Berlin, Germany) operated at 400.13 MHz for ^1^H and 100.63 MHz for ^13^C NMR procedures. All samples were dissolved in D_2_O and deuterium exchanged by successive lyophilization steps in D_2_O. 2D NMR analyses used the standard pulse programs of the instrument.

### 3.3. Biological Analyses

#### 3.3.1. Cells and Viruses

Cells: Vero cells (African green monkey kidney epithelial cells, ATCC CCL81) and *Aedes albopictus* cell clone C6/36 (ATCC CRL-1660) were cultured in Dulbecco’s modified Eagle’s medium (DMEM—Invitrogen-Gibco, Waltham, MA, USA) supplemented with 10% fetal bovine serum (Invitrogen-Gibco, Waltham, MA, USA), 2 mM glutamine (Sigma-Aldrich, St. Louis, MO, USA), and treated with 100 μg mL^−1^ streptomycin (Gibco BRL, Waltham, MA, USA), 100 IU mL^−1^ penicillin (Novafarma Ind. Farm., Anápolis-GO, Brazil), and 2.5 μg mL^−1^ amphotericin B (Meizler Biopharma S/A, Barueri-SP, Brazil). Vero and C6/36 cell cultures were maintained, respectively, at 37 °C and 28 °C with a 5% CO_2_ tension until a cell monolayer was obtained.

Viruses: Herpes simplex virus type 1 (HSV-1) strain KOS (ACV-sensitive) and AR-29 strain (ACV-resistant) were provided by the Departamento de Ciências Básicas da Saúde, Universidade Estadual de Maringá (Maringá-PR, Brazil). Dengue virus serotype 2 (DENV-2) was provided by Evandro Chagas Institute (Ananideua-PA, Brazil). Poliovirus (PV) was a Sabin strain. HSV-1 and PV stocks were obtained by inoculation in Vero cells and DENV-2, in C6/36 cells, until a cytopathologic effect was observed (about 90%), and aliquoted with glycerol (10%). The test aliquots were kept at −20 °C for a short period, and the stocks were stored at −80 °C. Titration was performed in Vero cells using a standard TCID50 (50% tissue culture infective dose) assay [41]. Serial (1:10) dilutions of viral stock suspension were prepared in microtiter tubes (450 µL of DMEM + 50 µL of viral suspension). Then, 100 µL of each dilution was added to the monolayer of Vero cells previously formed in 96-well plates. The plates were incubated at 37 °C (HSV and PV) and 28 °C (DENV), in 5% CO_2_ and examined daily under an inverted microscope to note the cytopathic effect. The 50% tissue culture infectious dose (TCID_50_) for each virus was calculated by the Reed and Muench method [41].

#### 3.3.2. Cytotoxicity Assay

The cytotoxicity of BOT derivatives was evaluated by the MTT method [42,43]. Confluent cultures (2 × 10^4^ cells/well) in 96-well microplates (Techno Plastic Products, Trasadingen, Switzerland) were exposed to different concentrations of the polysaccharides (100—6 µg mL^−1^). The MTT reagent (10 μL, final concentration of 1.25 mg mL^−1^) was added to each well, followed by an incubation period of 2 h at 37 °C, during which time the water-soluble MTT converts into insoluble formazan. The MTT solubilization solution (TritonX-100 + acidified isopropanol, 90 μL) was added to solubilize the formazan crystals. After vigorous shaking over 15 min, the absorbance was measured in a microplate reader (Bio-Tek Instruments, ELX800, Winooski, VT, USA) at 570 nm and 690 nm.

The percentage of cell viability (CV%) was calculated from the formula:

$$\mathrm{CV}\%=({\mathrm{At}}_{570\;\mathrm{nm}}-{\mathrm{At}}_{690\;\mathrm{nm}}/{\mathrm{Acc}}_{570\;\mathrm{nm}}-{\mathrm{Acc}}_{690\;\mathrm{nm}})\times 100$$

where At and Acc refer to the absorbance of test compounds and the control cells (untreated cells), respectively.

The 50% cytotoxic concentration (CC_50_) was calculated as the concentration of the derivatives required to reduce Vero cell viability by 50%, as determined by linear regression analysis.

#### 3.3.3. Evaluation of Anti-HSV, Anti-DENV, and Anti-PV Activities

The antiviral activity against HSV-1 strains KOS and AR, DENV-2, and PV was determined by TCID50 by reducing the viral titer [44,45] and MTT method [46], with minor modifications. Vero cells (1 × 10^4^ cells mL^−1^) were cultured in 96-well microplates for 24 h at 37 °C, under 5% CO_2_. The medium was removed, and 100 μL of a mixture containing the virus (multiplicity of infection of 1, MOI 1) and different concentrations of the tested derivatives (100 to 6 μg mL^−1^) were simultaneously added to the Vero cells. Cell (CC) and viral (VC) controls were analyzed by adding 100 μL of DMEM or viral suspension, respectively. The microplates were incubated for 72 h, under the same conditions of temperature and CO_2_, and the percentage of viral inhibition (VI) was calculated according to Sacchelli et al. (2019) [20].

The effective concentration that reduced the viral infection by 50% (IC_50_), compared to the cell and virus controls, was determined using linear regression analysis. The selectivity index (SI) was calculated from the ratio: CC_50_/IC_50_.

Statistical analysis ANOVA followed by Tukey’s test were applied to determine the difference among the compounds in the experiment and control groups. Values of *p* < 0.05 were considered significant.

## 4. Conclusions

The carboxymethylated and sulfonated-carboxymethylated derivatives of botryosphaeran with different degrees of substitution were prepared by chemical methods. All of the BOT derivatives displayed no cytotoxicity in Vero cells. While the carboxymethylated derivative was inactive against HSV, all sulfonated-carboxymethylated derivatives exerted remarkable inhibition against both HSV-KOS and HSV-AR strains, in a dose-dependent manner. The inhibitory potency of the present derivatives was slightly weaker than that observed for sulfonated botryosphaerans reported in our previous work [20]. Therefore, mimicking heparan sulfate with sulfonated-carboxymethylated β-glucan derivatives did not result in a better degree of inhibition of HSV than sulfonated β-glucan derivatives. However, as the antiherpetic activity of native BOT was enhanced by the derivatization procedure, the present work was successful in producing stronger antivirals.

## Figures and Tables

**Table 1:** The degrees of substitution (DSs) and antiviral activity of carboxymethylated BOT (CM-BOT) and its sulfonated derivatives (CM-S-BOT-1-3) against herpesvirus (KOS and AR strains).

			HSV-KOS		HSV-AR	
Compounds	DS_S_	^a^ CC_50_	^b^ IC_50_	^c^ SI	^b^ IC_50_	^c^ SI
BOT ^d^	0	>100	39.3	>2.5	47.5	>2.1
CM-BOT	0	>100	NIL	---	NIL	---
CM-S-BOT-1	0.1	>100	12.4	>8.0	56.0	>1.8
CM-S-BOT-2	0.2	>100	35.0	>2.8	40.0	>2.5
CM-S-BOT-3	0.4	>100	>100	---	25.7	>3.9
Acyclovir	0	>500	0.13	>3846	>8	>62.5

^a^ 50% cytotoxic concentration (μg mL^−1^). ^b^ 50% inhibitory concentration (μg mL^−1^). ^c^ Selectivity Index (CC_50_/IC_50_). ^d^ Sacchelli et al., 2019 [20]. Nil: no effect.

## Supplementary Materials

The following are available online at https://www.mdpi.com/article/10.3390/ijms222011013/s1.
