# Supplementary material for: Sulfonated and Carboxymethylated β-Glucan Derivatives with Inhibitory Activity against Herpes and Dengue Viruses
Source: Int J Mol Sci. 2021 Oct 12;22(20):11013. doi: 10.3390/ijms222011013 (PMC8538634; doi:10.3390/ijms222011013)
Supplement: Supplementary file 1 [file ijms-22-11013-s001.zip › ijms-1393025-supplementary.pdf]

Sulfonated and Carboxymethylated β-Glucan Derivatives with Inhibitory Activity against Herpes and Dengue Viruses

José Louzinho Lopes, Vinicius Seiki Takemura Quinteiro, Jéssica Wouk, Maria Laura Darido, Robert F. H. Dekker, Aneli M. Barbosa-Dekker, Václav Vetvicka, Mário A. A. Cunha, Ligia Carla Faccin-Galhardi and Alexandre Orsato *

SUPPLEMENTARY MATERIAL

Figure S1. Full ^{1}H-^{13}C HSQC spectrum of carboxymethylated botryosphaeran (CM-BOT).

Figure S2. Full ^{1}H-^{13}C HSQC spectrum of sulfonated/carboxymethylated botryosphaeran (CM-S-BOT-1).

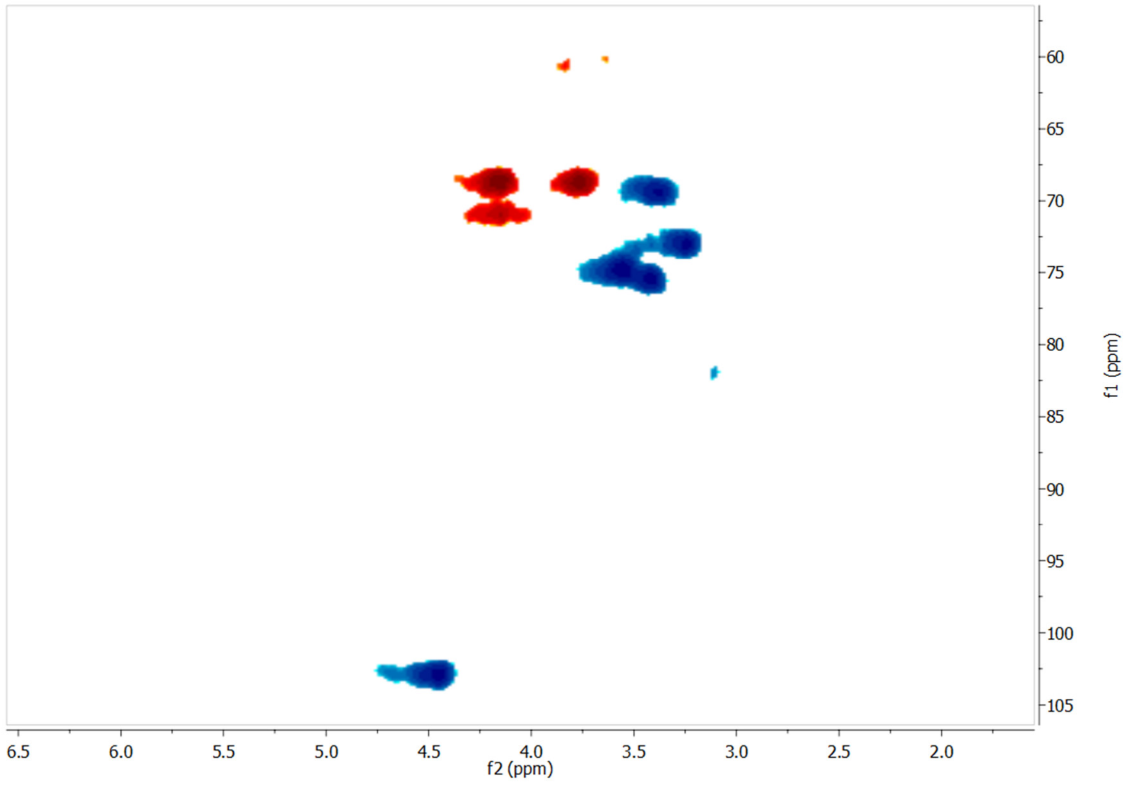

Figure S1. Full ^{1}H-^{13}C HSQC spectrum of carboxymethylated botryosphaeran (CM-BOT).

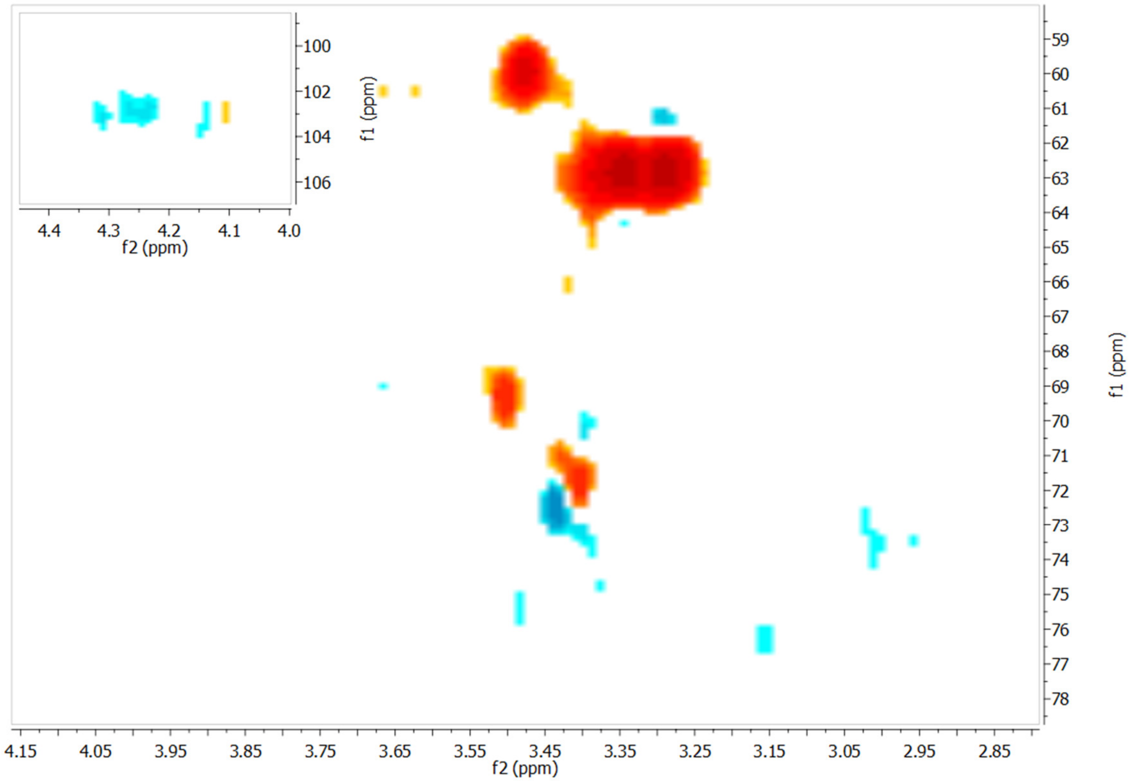

Figure S2. Full ^{1}H-^{13}C HSQC spectrum of sulfonated/carboxymethylated botryosphaeran (CM-S-BOT-1). Inset: anomeric region.
